# *Candida albicans*-Induced NETosis Is Independent of Peptidylarginine Deiminase 4

**DOI:** 10.3389/fimmu.2018.01573

**Published:** 2018-07-09

**Authors:** Eva Guiducci, Christina Lemberg, Noëmi Küng, Elisabeth Schraner, Alexandre P. A. Theocharides, Salomé LeibundGut-Landmann

**Affiliations:** ^1^Section of Immunology, Vetsuisse Faculty, University of Zurich, Zurich, Switzerland; ^2^Institute of Virology, Vetsuisse Faculty, University of Zurich, Zurich, Switzerland; ^3^Division of Hematology, University Hospital Zurich, University of Zurich, Zurich, Switzerland

**Keywords:** neutrophil extracellular traps, peptidylarginine deiminase 4, *Candida albicans*, host–fungus interaction

## Abstract

Neutrophils are the most abundant innate immune cells and the first line of defense against many pathogenic microbes, including the human fungal pathogen *Candida albicans*. Among the neutrophils’ arsenal of effector functions, neutrophil extracellular traps (NETs) are thought to be of particular importance for trapping and killing the large fungal filaments by means of their web-like structures that consist of chromatin fibers decorated with proteolytic enzymes and host defense proteins. Peptidylarginine deiminase 4 (PAD4)-mediated citrullination of histones in activated neutrophils correlates with chromatin decondensation and extrusion and is widely accepted to act as an integral process of NET induction (NETosis). However, the requirement of PAD4-mediated histone citrullination for NET release during *C. albicans* infection remains unclear. In this study, we show that although PAD4-dependent neutrophil histone citrullination is readily induced by *C. albicans*, PAD4 is dispensable for NETosis in response to the fungus and other common NET-inducing stimuli. Moreover, PAD4 is not required for antifungal immunity during mucosal and systemic *C. albicans* infection. Our results demonstrate that PAD4 is dispensable for *C. albicans*-induced NETosis, and they highlight the limitations of using histone citrullination as a marker for NETs and PAD4^−/−^ mice as a model of NET-deficiency.

## Introduction

*Candida albicans* is one of the most common etiological agents of opportunistic fungal infections that occur predominantly in immunocompromised individuals but can also affect healthy individuals, e.g. as a consequence of antibiotic therapy. *C. albicans*-mediated diseases range from mild superficial infections affecting the oral and vaginal mucosa to life-threatening bloodstream infections (1). Invasive candidiasis remains most difficult to diagnose and to treat and is thus associated with a high mortality rate. The development of clinical symptoms depends on both the host immune status and microbiota and on *C. albicans* virulence traits. Among those, *C. albicans* has the ability to reversibly switch between spherical yeast cells and large filaments called hyphae (2). While the yeast form is thought to be relevant for dissemination, hyphal growth is essential for tissue invasion and destruction (3). *C. albicans* filamentation is associated with increased expression of virulence factors, including adhesins, invasins, and hydrolytic enzymes that contribute to disrupting epithelial and endothelial barriers (4, 5). A robust immune response is required to protect the host against *C. albicans* infections (4, 6). Neutrophils are polymorphonuclear leukocytes of the innate immune system that constitute the first line of defense against *C. albicans* infections limiting fungal growth and dissemination (7). The high incidence of candidiasis in neutropenic patients as well as data from experimental studies in mice provide evidence for the critical protective role of neutrophils in antifungal defense (8, 9). Neutrophils can, however, also contribute to immunopathology and organ failure in some conditions (10, 11). Neutrophils are rapidly recruited from the bloodstream to the site of infection, where they engulf and kill pathogens by phagocytosis or *via* extracellular defense mechanisms (7, 12, 13). While *C. albicans* yeast cells are rapidly phagocytosed, hyphae are too large to be engulfed by neutrophils (14).

The release of neutrophil extracellular traps (NETs) has emerged as a prevalent strategy of neutrophils to combat large microbes (15). NETs are, as their name implies, web-like structures of chromosomal DNA decorated with histones and antimicrobial effector molecules (16), including granular proteins such as neutrophil elastase (NE), myeloperoxidase (MPO), and cytoplasmic proteins such as calprotectin (13). NETs have been widely shown to be induced by and to trap bacterial (12) and fungal pathogens (17) as well as protozoan parasites (18–20). However, NET formation or impaired NET degradation have also been shown to play a pathological role in non-infectious conditions, including the development of inflammatory and autoimmune diseases like systemic lupus erythematosus (21), thrombosis (22), autoimmune vasculitis (23), and psoriasis (24). Moreover, NETs were shown to be involved in metastatic processes, by trapping migrating tumor cells in the microvasculature and promoting the formation of micrometastasis (25).

Neutrophil extracellular trap release requires the production of reactive oxygen species (ROS) by NADPH oxidase (26). ROS promotes the translocation of NE from the azurophilic granules to the nucleus where it digests nucleosomal histones and promotes chromatin relaxation. Subsequently, MPO associates with chromatin and, synergistically with NE, promotes massive chromatin decondensation (27, 28). In association with diverse granular and cytoplasmic proteins (13), decondensed chromatin is eventually released into the extracellular space (28).

NETosis was further proposed to involve histone deimination by peptidylarginine deiminase 4 (PAD4) (29, 30). PAD4 is primarily expressed in the nucleus of neutrophils, where it catalyses the conversion of histone arginine to citrulline residues in a calcium-dependent manner (31). Citrullination is a posttranslational modification that plays an important role in many physiological processes, including skin keratinization, brain plasticity, gene regulation, and immune functions. Abnormal citrullination, though, can lead to the development of autoimmune diseases and cancer (32). Numerous stimuli have been reported to lead to PAD4 activation and NET formation (33); these include phorbol 12-myristate 13-acetate (PMA) (33), the calcium ionophore ionomycin (34), and various bacteria, viruses, and parasites (35).

Although PAD4-dependent histone citrullination is widely accepted as a key component in NETosis, firm evidence is still missing. Here, we use PAD4^−/−^ mice to assess the relevance of PAD4 in NETosis and in host protection against *C. albicans* infection.

## Materials and Methods

### Mice

PAD4^−/−^ mice on the C57BL/6 background (35) were obtained from late Kerri A. Mowen (La Jolla, CA, USA) and bred at the Laboratory Animal Service Center (University of Zürich, Switzerland). Wild-type (WT) C57BL/6J mice were purchased from Janvier Elevage. In some experiments, PAD4^+/+^ mice were used as WT, which were obtained by intercrossing PAD4^+/−^ mice. All mice were kept in specific pathogen-free conditions and used in sex- and age-matched groups at 6–12 weeks of age.

### Fungal Strain

The *C. albicans* strain SC5314 (36) was used for all experiments except where stated otherwise. The yeast-locked strain *hgc1*Δ/Δ and its revertant *hgc1*Δ/Δ:*HGC1* (37) were obtained from N. Gow (Aberdeen, UK). The oral isolate 101 (38) and strain pACT1-GFP (39) were described previously. All strains were grown in YPD medium at 30°C and 180 rpm for 15–18 h. For induction of hyphae, 5 × 10^3^ cfu *C. albicans* yeast cells were incubated in Hanks’ Balanced Salt Solution (HBSS; Life Technologies) containing CaCl_2_ and MgCl_2_ and supplemented with 5% FCS for 3 h at 37°C in a humidified atmosphere containing 5% CO_2_. In some experiments, *C. albicans* hyphae were opsonized for 30 min on ice in HBSS supplemented with 10% fresh mouse serum (for assays with murine neutrophils).

### Murine Infection Models

For systemic infection, mice were infected *via* the lateral tail vein with 1.5 × 10^5^ *C. albicans* yeast cells unless stated otherwise. For oropharyngeal candidiasis (OPC), mice were infected sublingually with 2.5 × 10^6^ *C. albicans* yeast cells as described (40), without immunosuppression. Mice were monitored for morbidity throughout the course of all experiments. For determination of the fungal burden, the kidneys (systemic candidiasis) or the tongue (OPC) of euthanized animals were removed, homogenized in sterile 0.05% NP40 in H_2_O for 3 min at 25 Hz using a Tissue Lyzer (Qiagen), and serial dilutions were plated on YPD agar containing 100 µg/ml ampicillin.

### Preparation of Tongue and Kidney Cells for Flow Cytometry Analysis

Mice were anesthetized with a sublethal dose of Ketamine (100 mg/kg), Xylazin (20 mg/kg), and Acepromazin (2.9 mg/kg) and perfused by injection of PBS into the right heart ventricle prior to removing the kidneys or the tongue. Kidneys were cut into fine pieces and digested with DNase I (200 µg/ml, Roche) and Collagenase I (240 mg/ml, Invitrogen) in RPMI at 37°C for 20 min. Tongues were cut into fine pieces and digested with DNase I (200 µg/ml, Roche) and Collagenase IV (4.8 mg/ml, Invitrogen) in PBS at 37°C for 45–60 min. Single cell suspensions were passed through a 70 µm strainer using ice-cold PBS supplemented with 1% FCS and 2 mM EDTA and analyzed by flow cytometry.

### Flow Cytometry

The single cell suspensions of kidneys and tongues were stained in ice-cold PBS supplemented with 1% FCS, 5 mM EDTA, and 0.02% NaN_3_ with LIVE/DEAD Fixable Near-IR Stain (Life Technologies). For staining of surface markers, cells were incubated with fluorochrome-conjugated antibodies against mouse CD45.2 (clone 104), CD11b (clone M1/70), Ly6G (clone 1A8); Ly6C (clone AL-21). All antibodies were from BioLegend. Stained cells were analyzed on a FACS LSRII (BD Biosciences) or on a FACS Gallios (Beckman Coulter), and the data were analyzed with FlowJo software (Tristar). Only live single cells were included in the analysis, and cell numbers were determined using fluorescent counting beads (BD Biosciences; Beckman Coulter).

### Histology and Immunohistochemistry of Mouse Tissue Sections

Mice were euthanized on the indicated day after infection and the kidneys or the tongue were removed, fixed in 4% PBS-buffered paraformaldehyde overnight, and embedded in paraffin. For histology, tissue sections (3–5 µm) were mounted on glass slides and were stained with Periodic-Acid Schiff (PAS) reagent and counterstained with hematoxylin. For immunohistochemical staining, a Dako Autostainer (Dako Autostainer Universal Staining System Model LV-1, Dako-Agilent Technologies) or the Discovery XT platform (Ventana), were used. Tissue sections on poly-l-lysine-coated glass slides were deparaffinized in xylene and rehydrated in a graded series of alcohol. Antigens were retrieved by incubation in citrate buffer, pH 6 at 98°C for 20 min. Sections were then incubated with rat anti-mouse Ly6G (clone 1A8, 5 µg/ml, BioLegend) or rabbit polyclonal anti-human citrullinated histone H3 (CitH3, 1 µg/ml, Abcam) for 60 min at room temperature (for anti-Ly6G) or at 37°C (for anti-CitH3). After blocking of endogenous peroxidases (Peroxidase blocking reagent; Dako) for 10 min at room temperature, slides were incubated for 30 min at room temperature with a detection antibody (biotinylated rabbit anti-rat IgG, 15 µg/ml, Vector, followed by avidin-horseradish peroxidase, Vector, for anti-Ly6G; or OmniMap anti-rabbit horseradish peroxidase, Ventana, for anti-CitH3), followed by incubation with 3,3-diaminobenzidine as chromogen and light counterstain with hematoxylin. Images were acquired with a digital slide scanner (NanoZoomer-XR C12000; Hamamatsu) and analyzed with the NDP.view2 software (Hamamatsu). The renal cortex and the tongue epithelium were defined based on anatomical criteria.

### RNA Isolation and Quantitative RT-PCR From Mouse Tissues

Isolation of total RNA from kidney tissues was carried out according to standard protocols using Trizol Reagent (Sigma). cDNA was generated by RevertAid reverse transcriptase (Thermo Scientific). Quantitative PCR was performed using SYBR Green (Roche) and a QuantStudio 7 Flex instrument (LifeTechnologies). The primers were *Kim-1* fwd 5-ATGAATCAGATTCAAGTCTTC-3′ and *Kim-1* rev 5-TCTGGTTTGTGAGTCCATGTG-3; *Actb* fwd 5-CCCTGAAGTACCCCATTGAAC-3′ and *Actb* rev 5- CTTTTCACGGTTGGCCTTAG-3. All qPCR assays were performed in duplicates and the relative gene expression (rel. expr.) was determined after normalization to β-actin transcript levels.

### Serological Determination of Renal Function

Blood was collected from infected mice by cardiac puncture and serum was obtained by centrifugation of the clotted blood for 90 s at 15,000 *g* and stored at −20°C until analysis. Blood urea nitrogen (BUN) and creatinine concentrations were determined using QuantiChrom™ Urea and QuantiChrom™ Creatinine assay kits (BioAssay Systems) according to the manufacturer’s instructions.

### Human Peripheral Blood Neutrophils

Peripheral blood was freshly drawn into EDTA-monovettes by venous puncture from two healthy adult volunteers and two patients with acquired MPO-deficiency. Samples were kept at room temperature during the entire isolation procedure. Neutrophils were freshly isolated over polymorphprep solution (Axis-Shield) by density centrifugation for 35 min at 450 *g*, without brake. The polymorphonuclear cell fraction was collected in a new tube and normal osmolarity was restored by adding an equal volume of 0.45% NaCl. To lyse contaminating erythrocytes, the cell pellet was resuspended in 3 ml of 0.2% NaCl for 30 s followed by addition of 3 ml of 1.6% NaCl and 4 ml PBS. The lysis was repeated 1–2 times until no erythrocytes were visible. Neutrophils were then resuspended in HBSS containing CaCl_2_ and MgCl_2_ and counted with trypan blue.

### Murine Bone Marrow Neutrophils

Mice were anesthetized with a sublethal dose of Ketamine (100 mg/kg), Xylazin (20 mg/kg), and Acepromazin (2.9 mg/kg) and perfused by injection of PBS into the right heart ventricle prior to remove the bones. The bone marrow was flushed from femurs and tibiae and passed through a 70 µm strainer using ice-cold PBS supplemented with 1% FCS and 2 mM EDTA. Neutrophils were isolated over a Histopaque-1077 and Histopaque-1119 gradient by density centrifugation at 710 *g* for 30 min, without brake. The polymorphonuclear cell fraction was collected in HBSS containing CaCl_2_ and MgCl_2_. Erythrocytes were lysed using erythrocytes lysis buffer (0.3 M NH_4_Cl, 28 µM NaHCO_3_, 125 µM EDTA). Neutrophil purity was assessed by flow cytometry by staining for CD45^+^ CD11b^+^ Ly6G^+^ neutrophils and was found to be generally between 70 and 80%. The cell numbers used in each experiment were adjusted accordingly to guarantee that equal numbers of neutrophils were present in all experimental groups (e.g., WT vs. PAD4^−/−^ neutrophils).

### Quantification of NET Release From Activated Neutrophils

Freshly isolated human and mouse neutrophils (10^5^ cells) were cultured in 96-well tissue culture plates in HBSS containing CaCl_2_ and MgCl_2_ and stimulated with PMA (100 ng/ml, Sigma-Aldrich), Ionomycin (1 µM, Sigma-Aldrich), or with 5 × 10^3^ *C. albicans* yeast or preformed hyphae for 2.5 h at 37°C. Human neutrophils were treated with the PAD inhibitor Cl-amidine (200 µM, Merck Millipore) for 2.5 h. The activity of Cl-amidine was confirmed by means of CitH3 staining. After the incubation, Sytox Green (160 nM, Invitrogen) was added to the cells for detection of extracellular DNA. Unstimulated neutrophils were used as controls. The plates were analyzed on an Infinite 200 plate reader (Tecan) with excitation at 485 nm and emission at 535 nm. The fluorescence of stimulated cells was calculated by subtracting the baseline fluorescence of unstimulated cells and is expressed in arbitrary units. In some experiments, Sytox Green staining was also analyzed by fluorescence microscopy.

### Immunofluorescence Staining

10^5^ isolated human or mouse neutrophils were seeded on glass coverslips in tissue-culture plates and incubated with PMA (100 ng/ml, Sigma-Aldrich), Ionomycin (1 µM, Sigma-Aldrich), or 5 × 10^3^ *C. albicans* preformed hyphae for 2.5 h at 37°C. Cells were then fixed with 4% paraformaldehyde, permeabilized with 0.01% TritonX-100 for 10 min, and blocked with 5% donkey serum for 30 min at room temperature. Cells were stained with goat polyclonal anti-MPO (R&D systems), rabbit polyclonal anti-CitH3 (Abcam), and combined rat anti-S100A8 and anti-S100A9 (clone 10 and clone 46 respectively, kindly provided by C. Urban, Umeå, Sweden). Secondary antibodies were donkey anti-goat IgG (Jackson ImmunoResearch), goat anti-rat IgG (Abcam), and goat anti-rabbit IgG (Jackson ImmunoResearch). The PKH26 Red Fluorescent Cell Linker Kit (Sigma-Aldrich) was used to stain lipids of the neutrophil plasma membranes. DNA was stained with 4′,6′-Diamidino-2-phenylindole dihydrochloride (DAPI, Sigma-Aldrich) and NETs were visualized using a Leica SP8 inverse confocal microscope and analyzed with FIJI software.

### Scanning Electron Microscopy

Freshly purified murine bone marrow neutrophils were stimulated on glass objective slides in a 24-well cell culture plate with preformed *C. albicans* hyphae, 100 ng/ml PMA, and 1 µM ionomycin in HBSS for 2.5 h at 37°C and 5% CO_2_, then, cells were fixed with 0.1 M Cacodylate buffer (Merck) containing 2.5% glutaraldehyde. After washing three times with PBS, the samples were treated with 1% Osmiumtetroxide in PBS for 30 min, washed again three times with PBS, dehydrated by incubation in 70% EtOH in H_2_O for 30 min followed by 100% EtOH for 30 min and an incubation with hexamethyldisilazane (Sigma) for 2 h prior to air drying overnight. The specimen surfaces were sputter coated with 4 nm gold/palladium (CCU-010 HV, Safematic, Switzerland) and imaged using a Zeiss Supra 50 VP scanning electron microscope using the secondary electron detector (Zeiss, Oberkochen, Germany).

### Transmission Electron Microscopy

4 × 10^6^ bone marrow neutrophils were incubated with 5 × 10^5^ cfu *C. albicans* preformed hyphae for 2.5 h at 37°C. Cell suspensions were centrifuged for 5 min at 5.9 *g*, and the supernatants were discarded. Pellets were re-suspended with 2.5% glutaraldehyde in 0.1 M Na/K-phosphate, transferred to a micro tube, and centrifuged for 20 min at 3,400 *g*. The pellets were rinsed with 0.1 M Na/K-phosphate and postfixed with 1% osmium tetroxide in 0.1 M Na/K-phosphate for 1 h, dehydrated in a series of ethanol starting at 70%, and after transferring into acetone embedded in epon followed by polymerization at 60°C for 2.5 days. Sections of 60–80 nm thickness were stained with uranyl acetate and lead citrate and analyzed in a transmission electron microscope (CM12, FEI, Eindhoven, The Netherland) equipped with CCD cameras (Ultrascan 1000 and Orius SC1000, Gatan, Pleasanton, CA, USA) at an acceleration voltage of 100 kV.

### *C. albicans* Killing by Neutrophils

5 × 10^4^ neutrophils were added to each well of a 96-well microplate containing 5 × 10^3^ *C. albicans* preformed hyphae and incubated for 3 h at 37°C. The neutrophils were then lysed with water supplemented with 0.02% Triton-X-100, and the metabolic activity of *C. albicans* was assessed with Alamar blue (Invitrogen) according to the manufacturer’s instructions. A standard curve was generated with serial dilutions of hyphal cells (without neutrophils). Killing activity is expressed as percent of viable fungi in the presence of neutrophils compared to the conditions without neutrophils.

### *C. albicans* Uptake by Neutrophils

2.5 × 10^4^ murine bone marrow neutrophils were incubated for 1 h at 37°C with either opsonized or non-opsonized pACT1-GFP *C. albicans* yeast cells at a fungus:neutrophil ratio of 1:1. After washing, neutrophils were incubated with anti-Ly6G antibody (BioLegend) for 20 min at 4°C. The degree of *C. albicans* uptake was determined by counting the number of GFP^+^ neutrophils relative to the total number of neutrophils by flow cytometry.

### ROS Assay

2 × 10^5^ mouse neutrophils were added to each well of a 96-well microtiterplate containing 10^5^ *C. albicans* yeast cells or preformed hyphae. Total ROS production was measured by adding cell-permeable luminol (100 µM, Sigma-Aldrich), while extracellular ROS was assessed by adding cell-impermeable lucigenin (400 µM, Sigma-Aldrich). Chemiluminescence was measured on an Infinite 200 plate reader (Tecan) every 2.5 min over a total period of 2.5 h starting immediately after addition of the substrates.

### Statistics

Statistical significance was determined by unpaired *t* test with Welch’s correction or two-way ANOVA using GraphPad Prism (GraphPad Software) with *p < 0.05; **p < 0.01; ***p < 0.001. Data displayed on a logarithmic scale were log-transformed before statistical analysis.

## Results

### *C. albicans*-Induced Citrullination of Histone H3 Is Strictly Dependent on PAD4

**Figure 1:**
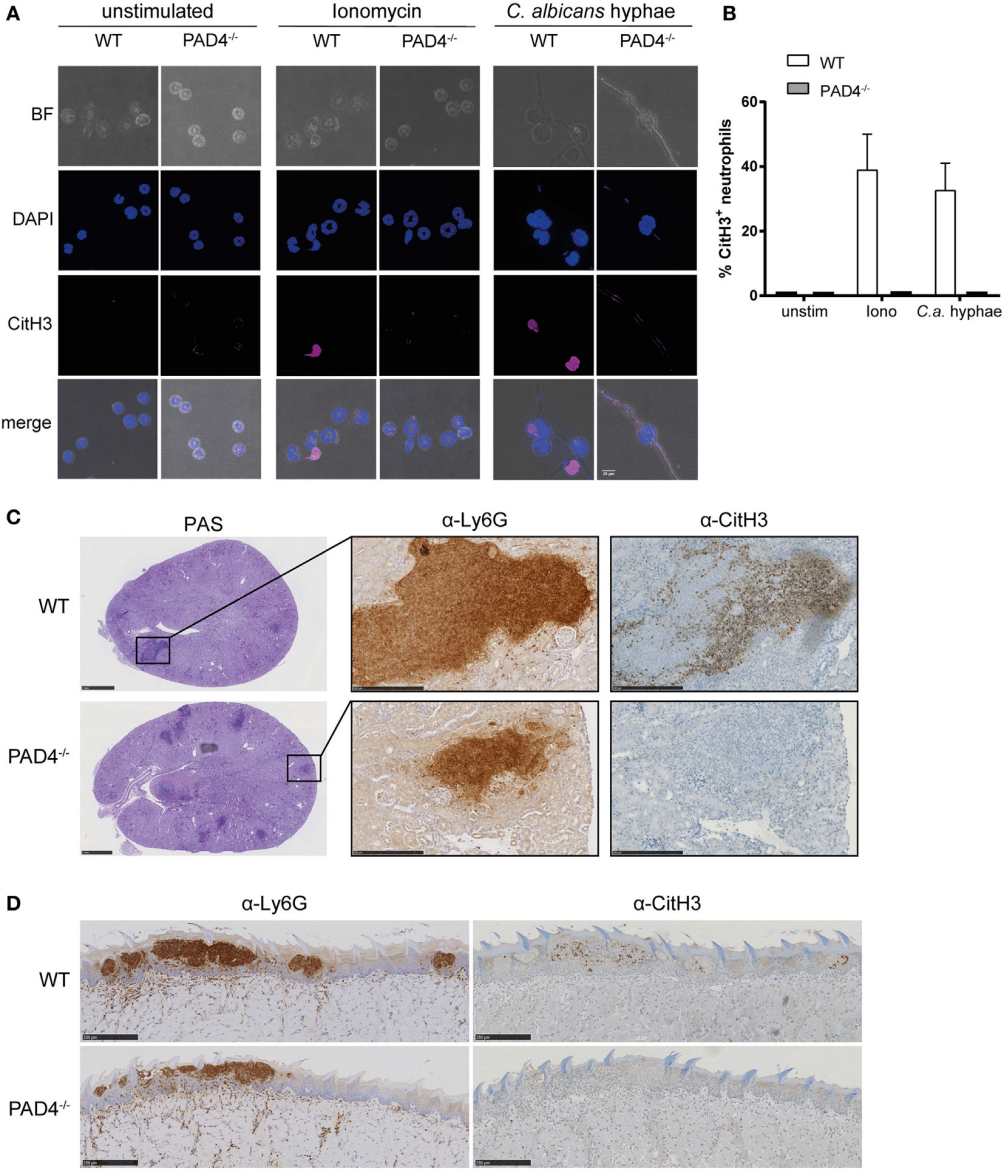
*Candida albicans* induces peptidylarginine deiminase 4 (PAD4)-mediated citrullination of histone H3. **(A,B)** Bone marrow neutrophils from WT and PAD4^−/−^ mice were stimulated for 2.5 h with *C. albicans* hyphae or ionomycin or left unstimulated and then stained for DNA (DAPI; blue) and citrullinated histone H3 (magenta); brightfield. Images were acquired by confocal microscopy **(A)** and the fraction of CitH3^+^ neutrophils was determined **(B)**. In **(B)**, the bars are the mean with standard error of the mean (SEM) of 10–30 cells analyzed per condition. **(C,D)** WT and PAD4^−/−^ mice were infected with *C. albicans via* the tail vein **(C)** or sublingually **(D)**. Consecutive kidney **(C)** or tongue **(D)** sections were stained with periodic-acid schiff reagent **(C)**, α-Ly6G or with α-CitH3 on day 3 postinfection **(C)** or on day 1 postinfection **(D)**, respectively. Representative images are shown. Scale bar = 50 µm [in **(A)**], 1 mm [in **(C)**, left panel], 250 µm [in **(C)** middle and right panel and in **(D)**]. See also Figure S1 in Supplementary Material.

We set out to assess the activity of PAD4 in response to *C. albicans*. Citrullination of histone H3 (CitH3) was strongly induced in murine bone marrow neutrophils when stimulated with *C. albicans* hyphae or with ionomycin as a control (Figures 1A,B). Notably, only a fraction of neutrophils acquired the modification with both stimuli. As expected, citrullination was completely absent in neutrophils lacking PAD4 due to a genetic deletion (Figures 1A,B).

Citrullination of histone H3 in response to *C. albicans* was also observed *in vivo* in the kidney of mice that were infected *via* the intravenous route with strain SC5314 (Figure 1C). The kidney is the primary target organ of the fungus in this model of infection, where *C. albicans* hyphae spread throughout the renal cortex and eventually extend to the renal tubules and pelvis (41). Neutrophils, and to some extent also inflammatory monocytes, infiltrate the infected kidney and accumulate around *C. albicans* hyphae forming dense inflammatory abscesses. Induction of CitH3 in the renal cortex of WT C57BL/6 mice occurred as early as 24 h postinfection and further increased over time (Figure S1A in Supplementary Material). Notably, only a fraction of all Ly6G-positive neutrophils stained positive for CitH3 (Figure S1B in Supplementary Material). As expected, CitH3 was absent in PAD4-deficient mice (Figure 1C).

Neutrophils are also implicated in antifungal defense in barrier tissues (42–44). In a model of OPC with *C. albicans* strain SC5314, neutrophils rapidly accumulate at sites where *C. albicans* hyphae invade the keratinized epithelium. They play a crucial role in preventing fungal dissemination and overgrowth within the first days of infection (43). We detected CitH3 to be induced as early as 18 h postinfection with a peak around 24 h postinfection (Figure S1C in Supplementary Material). Similarly to what we observed during systemic candidiasis, only a small fraction of all Ly6G-positive neutrophils adopted the posttranslational modification (Figure S1D in Supplementary Material) and citrullination of histone H3 was strictly dependent on PAD4 (Figure 1D).

### NETosis in Response to *C. albicans* Hyphae Is Independent of PAD4

**Figure 2:**
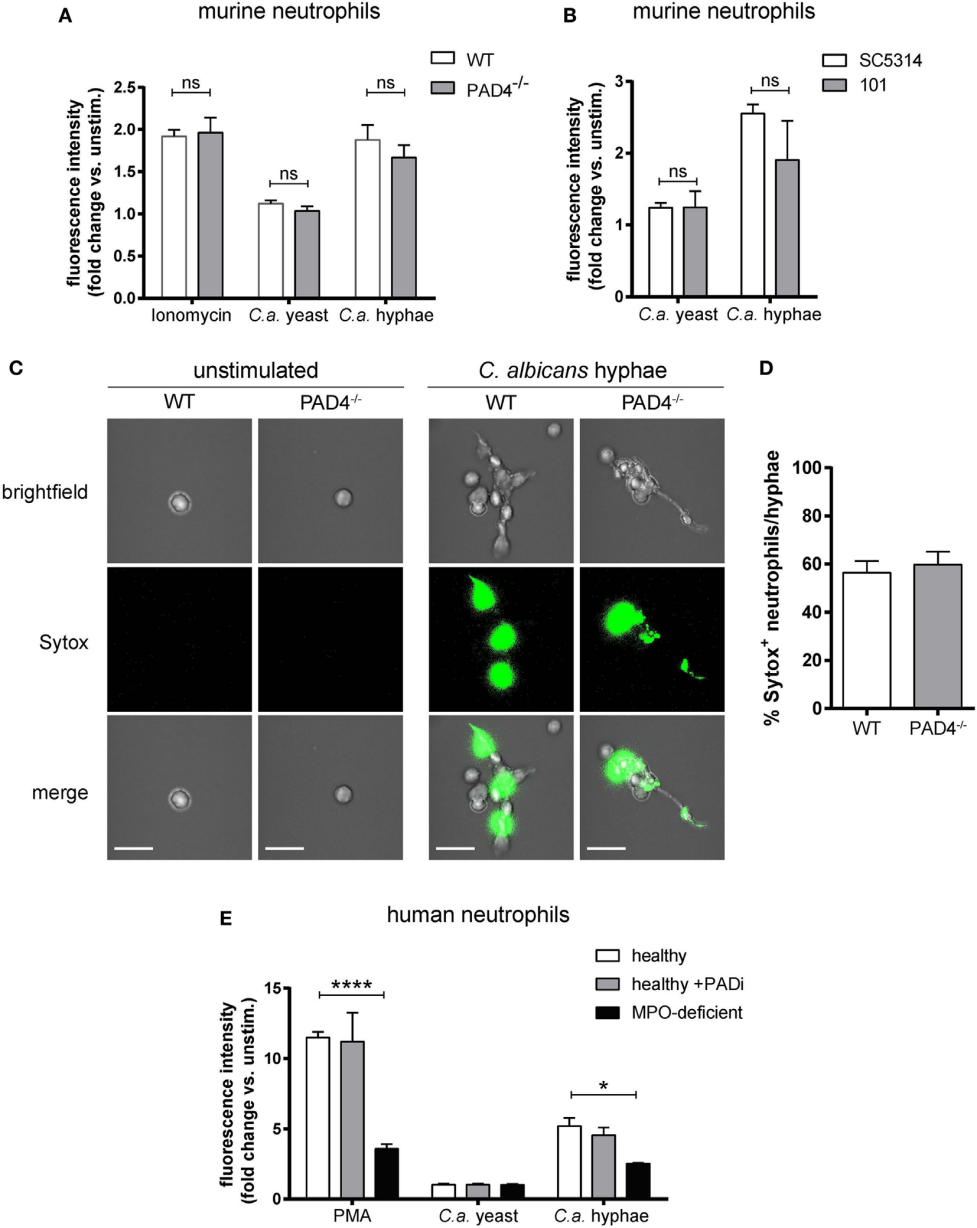
NETosis in response to *Candida albicans* hyphae is independent of Peptidylarginine deiminase 4 (PAD4). **(A)** The release of extracellular DNA from murine bone marrow neutrophils was detected by Sytox green after stimulation for 2.5 h with the yeast-locked strain Δ*hgc1* (*C.a*. yeast), preformed hyphae of the control strain Δ*hgc1* + HGC1 (*C.a*. hyphae), or ionomycin as indicated. The increase in fluorescence intensity from stimulated relative to unstimulated neutrophils is shown. Each bar represents the mean with standard error of the mean (SEM) of each group (*n* = 3) with data pooled from three independent experiments. **(B)** Bone marrow neutrophils were stimulated with *C. albicans* yeast cells or preformed hyphae of the highly virulent lab strain SC5413 and the low-virulent strain 101 as indicated. The release of extracellular DNA was detected by Sytox green as in **(A)**. Data are the mean with SEM of each group (*n* = 3) with data pooled from three independent experiments. **(C,D)** The release of DNA from WT and PAD4^−/−^ neutrophils that were stimulated and stained with Sytox as described in **(A)** was visualized by immunofluorescence microscopy. Representative images for each condition are shown in **(C)**. Scale bar = 20 µm. The frequency of Sytox^+^ neutrophils among all *C. albicans* hyphae-associated neutrophils is shown in **(D)**. The bars are the mean with SEM of 15 images analyzed per condition. **(E)** Human peripheral blood neutrophils from healthy donors that were treated with the PAD inhibitor Cl-amidine or left untreated and from patients with acquired myeloperoxidase-deficiency were stimulated for 2.5 h with the yeast-locked strain Δ*hgc1* (*C.a*. yeast), preformed hyphae of the control strain Δ*hgc1* + HGC1 (*C.a*. hyphae), or phorbol 12-myristate 13-acetate. Sytox was detected as described in **(A)**. Each bar represents the mean with SEM of three technical replicates of each group. Data are representative of one out of two independent experiments.

Next, we examined the role of PAD4 in NET formation in response to *C. albicans* hyphae. For this, we first characterized *C. albicans*-induced NETs in neutrophils that were isolated from the bone marrow of WT mice and stimulated with yeast cells (using the yeast-locked strain Δ*hgc1*) or with preformed hyphae (using the control strain Δ*hgc1* + HGC1). NET induction was quantified by Sytox green, a DNA-intercalating dye that is impermeant to live cells (45). *C. albicans* hyphae triggered a strong response, which was comparable to that of ionomycin-stimulated neutrophils, while no response was observed with *C. albicans* yeast cells (Figure 2A). *C. albicans* hyphae-induced NETosis was independent of fungal virulence, as both, the low-virulent strain 101 (38) and the highly virulent lab strain SC5413 triggered a comparable response (Figure 2B). To confirm that the increase in Sytox fluorescence in our assay indeed corresponded to NETotic neutrophils, we stained mouse neutrophils with Sytox green for inspection of extracellular DNA by fluorescence microscopy. While unstimulated neutrophils did not bind Sytox, *C. albicans* hyphae induced a strong Sytox signal and Sytox^+^ stretches of DNA extending from the cell body were observed (Figure 2C).

To assess the involvement of PAD4 in NETosis, we compared the capacity of PAD4-deficient and -sufficient neutrophils to induce NETs. Surprisingly, PAD4-deficiency did not affect the release of DNA from murine neutrophils in response to any of the stimuli tested (Figures 2A,C,D). The same was also true for human neutrophils that were treated with Cl-amidine to inhibit PAD activity in response to *C. albicans* hyphae or PMA (Figure 2E). As a control, we included a condition with neutrophils from patients with acquired MPO-deficiency (46). As expected, these cells were strongly impaired in their capacity to release NETs in response to *C. ablicans* hyphae or PMA (Figure 2E).

**Figure 3:**
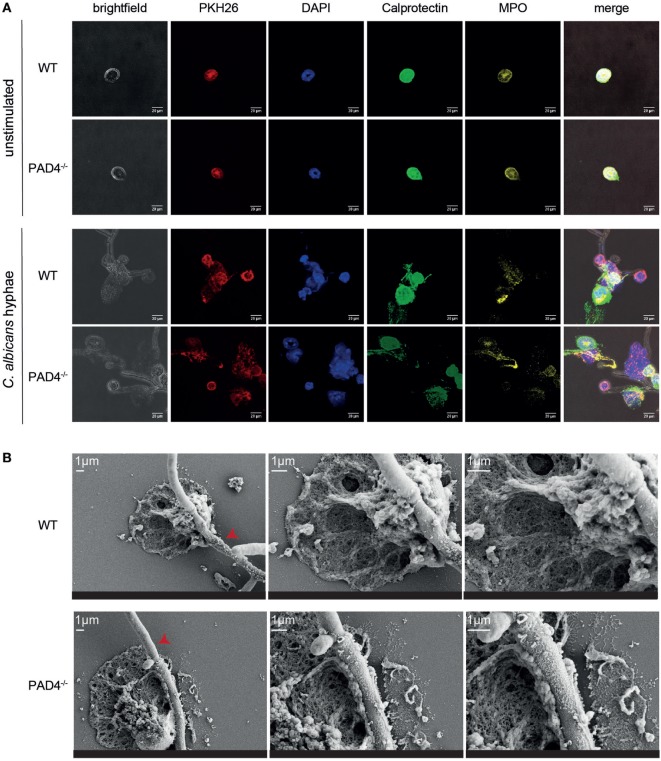
Characterization of neutrophil extracellular traps released from WT and PAD4^−/−^ neutrophils. **(A)** Bone marrow neutrophils isolated from WT and PAD4^−/−^ mice were stimulated for 2.5 h with *Candida albicans* hyphae or left unstimulated and then stained for membrane lipids (PKH26; red), DNA (DAPI; blue), calprotectin (anti-S100A8 and anti-S100A9, green), and myeloperoxidase (yellow). Images were acquired by confocal microscopy. Representative images of each group are shown. Scale bar = 20 µm. **(B)** Bone marrow neutrophils isolated from WT and PAD4^−/−^ mice were stimulated for 2.5 h with *C. albicans* hyphae and phorbol 12-myristate 13-acetate/ionomycin. Cells were fixed and imaged by scanning electron microscopy. Representative images of each group are shown. Scale bar = 1 µm. Red arrowheads show *C. albicans* hyphae.

We further compared NET release by WT and PAD4-deficient neutrophils by immunofluorescence microscopy. Upon stimulation of murine bone marrow neutrophils with *C. albicans* hyphae, we observed morphological changes in the plasma membrane of the cells (visualized by PKH26 staining), chromatin decondensation, and nuclear expansion characteristic of NETotic neutrophils (Figure 3A). Moreover, we found calprotectin and MPO, which are usually located intracellularly, to colocalize with extracellular DNA (labeled with DAPI, Figure 3A). Finally, we performed scanning electron microscopy with WT and PAD4^−/−^ neutrophils and found NETs in proximity of *C. albicans* hyphae to be indistinguishable between both genotypes (Figure 3B).

In line with our previous results, we could not detect any difference in the NETs released from PAD4^−/−^ neutrophils compared to their WT counterparts (Figure 3). Together, these data confirm that PAD4-mediated histone citrullination is dispensable for NETosis by both human and murine neutrophils in response to *C. albicans* hyphae.

### PAD4-Deficiency Results in Increased Clustering of Neutrophils Around *C. albicans* Hyphae but Does Not Affect Their Antifungal Activity

**Figure 4:**
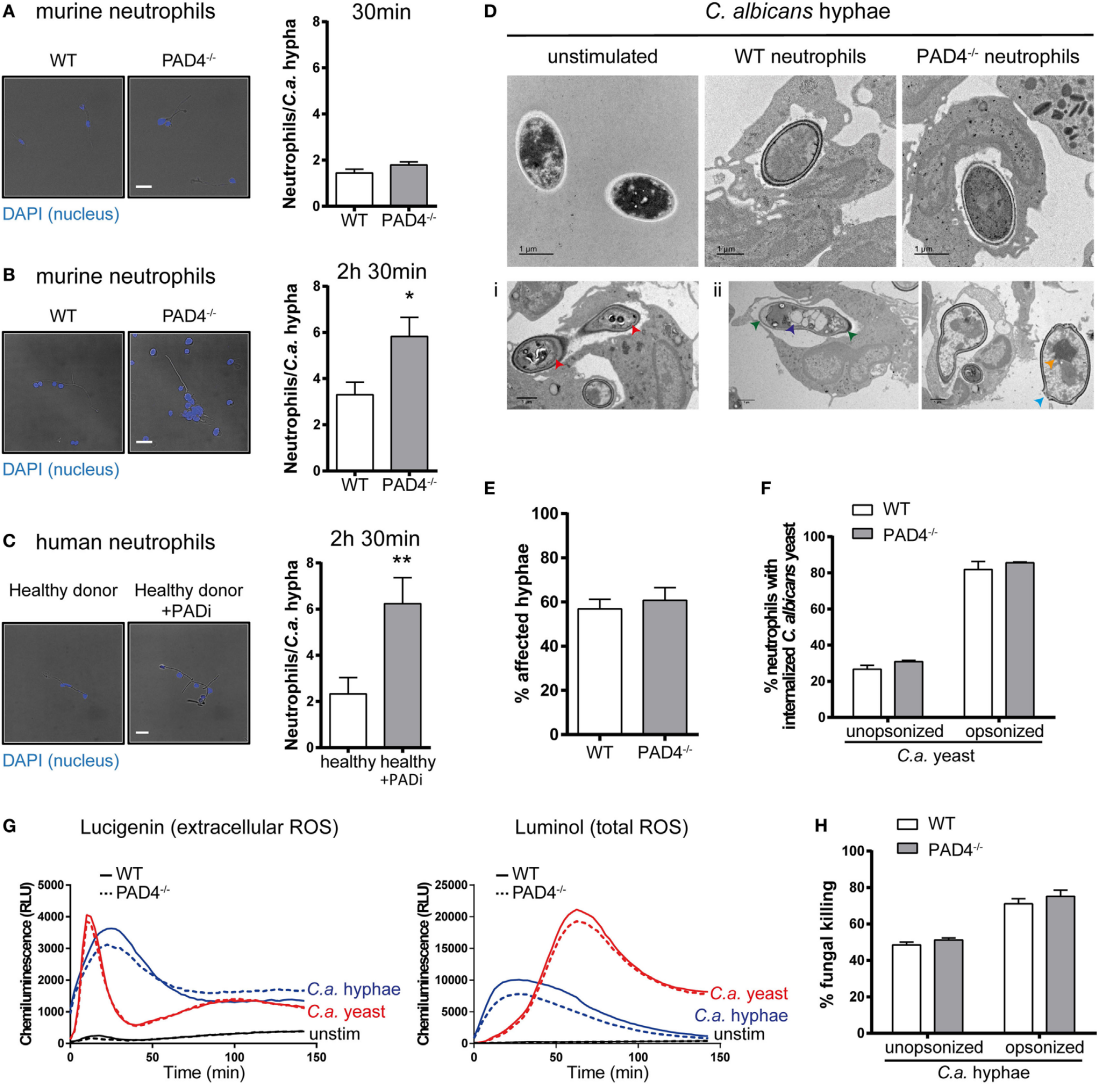
Peptidylarginine deiminase 4 (PAD4)-deficiency results in increased clustering of neutrophils to *Candida albicans* hyphae but does not affect their antifungal activity. **(A–C)** Neutrophils were isolated from the bone marrow of naïve WT and PAD4^−/−^ mice **(A,B)** or from peripheral blood of a healthy donor and treated or not with the PAD inhibitor Cl-amidine **(C)**. Neutrophils were incubated with *C. albicans* hyphae and then stained with DAPI (blue). The number of neutrophils interacting with each *C. albicans* hypha was quantified after 30 min **(A)** or 2.5 h **(B,C)** of stimulation. Representative images are shown on the left (Scale bar = 20 µm), summary plots are shown on the right. Bars are the mean with standard error of the mean (SEM) of each group with *n* = 2–3. **(D)** The cell wall integrity of *C. albicans* hyphae was assessed by transmission electron microscopy after 2.5 h incubation with WT or PAD4^−/−^ bone marrow neutrophils. Representative images of each group are shown in the upper row. Lower row: some hyphae exposed to neutrophils showed membrane retraction and extensive cytoplasm disintegration **(Di)** or vacuolation, nuclear alterations, and swelling of the cell wall **(Dii)**. Scale bar = 1 µm. **(E)** The frequency of *C. albicans* hyphae that were affected by WT and PAD4^−/−^ neutrophils as described in **(Di,Dii)** was quantified. Bars are the mean with SEM of fungal elements from 18 EM images analyzed per condition. **(F)** WT and PAD4^−/−^ bone marrow neutrophils were incubated with GFP-expressing *C. albicans* yeast cells and the degree of *Candida* uptake was determined by flow cytometry. Bars are the mean with standard error of the mean (SEM) of each group with *n* = 2. **(G)** Reactive oxygen species (ROS) production by WT and PAD4^−/−^ bone marrow neutrophils in response to *C. albicans* yeast or hyphae was detected by chemiluminescence using the cell-impermeable substrate lucigenin to detect extracellular ROS (left) or the cell-permeable substrate luminol to detect total ROS (right). **(H)** WT and PAD4^−/−^ bone marrow neutrophils were incubated for 3 h with opsonized or unopsonized *C. albicans* hyphae. The percentage of *C. albicans* killing was assessed by alamar blue assay. Bars are the mean with SEM of each group with *n* = 4. Data are pooled from **(A,B,H)** or representative of **(G)** two independent experiments.

Next, we investigated whether PAD4-deficiency may impact the activity of neutrophils in response to *C. albicans* hyphae by other means than NETosis. We observed clustering of neutrophils around fungal hyphae by fluorescence microscopy imaging. At 30 min of incubation, the number of neutrophils per fungal element was comparable for PAD4-deficient and -sufficient neutrophils with ~2 neutrophils per hypha (Figure 4A). At a later time point (2.5 h), however, the number of PAD4^−/−^ neutrophils per hypha was increased twofold compared to WT neutrophils (Figure 4B). This result was reproducible when performing the same type of analysis with human neutrophils: inhibition of PAD4 activity by Cl-amidine resulted in increased neutrophil numbers attached per *C. albicans* hyphal element (Figure 4C). We hypothesized that the increased clustering of PAD4^−/−^ neutrophils may be a consequence of an impaired ability of these cells to combat the fungus by surrounding it more densely. We, therefore, assessed by transmission electron microscopy how WT and PAD4^−/−^ neutrophils affect the fungal cell wall integrity. Exposure of *C. albicans* hyphae to WT neutrophils caused an increase in electron density in the cell wall, compared to free *C. albicans* hyphae (Figure 4D). The presence of metal ions, such as copper, results in higher electron density, which is visible as dark areas in transmission electron microscopy. Thus, the increase in electron density in the cell wall may be due to enhanced expression of the copper-dependent superoxide dismutase SOD5 in *C. albicans* upon exposure to neutrophils (47). We observed that some hyphae displayed membrane retraction and extensive cytoplasm disintegration when incubated with neutrophils (Figure 4Di), while others presented vacuolation, nuclear alterations, and swelling of the cell wall (Figure 4Dii). We quantified the frequency of such alterations in *C. albicans* hyphae in response to WT and PAD4^−/−^ neutrophils. However, we did not find any evidence for a difference between the two genotypes (Figure 4E).

Likewise, PAD4-deficiency did not impair the antifungal activity of neutrophils against *C. albicans*. PAD4^−/−^ neutrophils displayed normal capacity to phagocytose *C. albicans* yeast cells (Figure 4F) and to produce ROS in response to *C. albicans* yeast and hyphae (Figure 4G). Moreover, the ability to kill *C. albicans* was comparable between PAD4^−/−^ and WT neutrophils (Figure 4H). Collectively, these results indicate that PAD4-deficiency does not affect the antifungal activity of neutrophils *in vitro*. The meaning of the observed difference in neutrophil clustering around *C. albicans* hyphae could not be revealed by any of the assays performed so far, but we hypothesize that it may result in altered fungal control *in vivo*.

### PAD4 Is Not Essential for Fungal Control During Systemic and Mucosal *C. albicans* Infection

**Figure 5:**
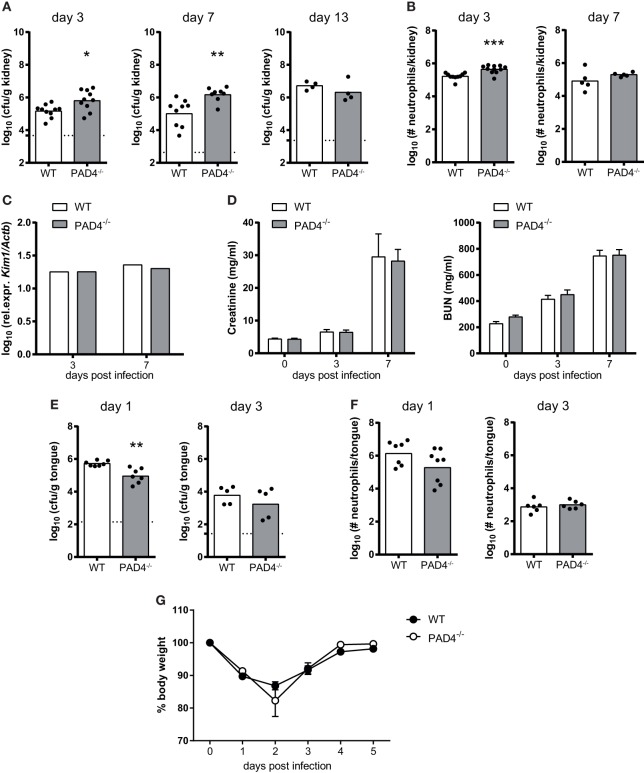
The role of peptidylarginine deiminase 4 (PAD4) during systemic and oropharyngeal candidiasis. **(A–D)** WT and PAD4^−/−^ mice were infected with *Candida albicans via* the tail vein. **(A)** The kidney fungal burden was determined on day 3, day 7, and day 13 postinfection. **(B)** CD45^+^ Ly6C^int^ Ly6G^+^ neutrophils were quantified in the kidney by flow cytometry on day 3 and day 7 postinfection. **(C)** Kidney injury molecule-1 (KIM-1) transcripts were quantified in kidney homogenates by qRT-PCR on day 3 and day 7 postinfection. **(D)** Creatinine and blood urea nitrogen levels were determined in the serum on day 3 and day 7 postinfection. **(E–G)** WT and PAD4^−/−^ mice were infected with *C. albicans* sublingually. **(E)** The tongue fungal burden was determined on day 1 and day 3 postinfection. **(F)** CD45^+^ Ly6C^int^ Ly6G^+^ neutrophils were quantified in the tongue by flow cytometry on day 1 and day 3 postinfection. **(G)** The body weight was monitored during the course of infection. In **(A,B,E,F)**, each symbol represents an individual mouse. The geometric mean **(A–C,E,F)** of each group is indicated. In **(D)**, the bars are the mean with standard error of the mean (SEM) of each group (*n* = 8–10). Data are pooled from two to three independent experiments. The dotted lines in **(A,E)** represent the detection limit. See also Figures S2 and S3 in Supplementary Material.

To assess whether PAD4-deficiency affects fungal control *in vivo*, we intravenously infected PAD4^−/−^ mice with *C. albicans*. The kidney fungal burden was slightly increased in PAD4^−/−^ mice compared to their WT counterparts, both on day 3 and day 7 postinfection (Figure 5A; Figure S2 in Supplementary Material). The increase in fungal load was accompanied by a slight increase in the number of kidney-infiltrating neutrophils (Figure 5B). However, the difference in PAD4^−/−^ mice compared to controls was transient, as similar fungal loads were found in the kidney of both mouse strains at a later time point of infection (Figure 5A) and the neutrophil infiltrates also swiftly normalized after the initial increase in PAD4^−/−^ mice (Figure 5B). This reversion of the initial defect in fungal control may possibly be explained by a compensatory effect of the adaptive immune system beyond 1 week postinfection. No differences in fungal load were observed in any of the other organs that we analyzed, including brain, liver, and spleen, nor when a higher or a lower infection dose was employed (Figure S3 in Supplementary Material). We also measured kidney injury molecule-1 (KIM-1) expression and creatinine and BUN levels in *C. albicans*-infected mice as indicators of immunopathology. No signs of altered kidney damage (Figure 5C) or renal dysfunction (Figure 5D) were detected in PAD4^−/−^ mice compared to WT controls.

We also addressed the relevance of PAD4 in antifungal defense during mucosal infection. PAD4^−/−^ mice displayed normal control of the fungus in the oral cavity and tongue fungal counts were even slightly reduced at an early time point of infection compared to WT controls (Figure 5E). Neutrophil recruitment to the site of infection was not affected (Figure 5F; Figure S2 in Supplementary Material) and weight recovery after infection (Figure 5G) was also normal in PAD4^−/−^ mice. These data indicate that PAD4 is not required for host protection against OPC, and that it has only a small and transient effect on the course of infection during systemic candidiasis.

## Discussion

Neutrophil extracellular traps were first described in 2004 by Brinkmann et al. as a novel mechanism used by neutrophils to combat infection by trapping and killing extracellular disease-causing organisms including large fungal particles such as *C. albicans* hyphae (12, 17). Since then, many examples have been reported where NETs contribute to host defense and/or promote pathologies ranging from sepsis, autoinflammatory and metabolic diseases to cancer (48). NETs are released by a programed form of cell death characterized by chromatin decondensation, disassembly of the nuclear envelope, and plasma membrane disruption. NADPH oxidase, NE, and MPO play a central role in coordinating these processes (49). The protein-arginine deiminase PAD4 was also implicated in NETosis *via* histone citrullination and chromatin decondensation (50). In this study, we report that despite the strong induction of PAD4-dependent histone citrullination by *C. albicans*, this pathway is dispensable for NETosis in both mouse and human neutrophils in response to fungal hyphae. Likewise, PAD4-deficiency resulted in at best a minor and transient defect in fungal control *in vivo*. This was consistent with normal antifungal effector functions of PAD4^−/−^ neutrophils, including phagocytosis and ROS production, as previously reported (30, 34). Thus, our data challenge the paradigm that PAD4-mediated histone citrullination is essential for chromatin decondensation during NETosis.

Many activating stimuli of neutrophils can induce histone citrullination (15, 29, 30, 51, 52). The observation that histone citrullination leads to chromatin decondensation associated with the release of nuclear DNA (29) led to the assumption that PAD4 activity is an integral part of the NETotic process (50). However, the requirement of PAD4 in NETosis was never formally proven and although the link between histone citrullination and NETosis is widely accepted it remains at best correlative. The excitement about the availability of an allegedly specific marker of NETosis has drowned critical voices questioning the erroneous concept of citrullination in NETosis (33, 53, 54). Our study now demonstrates that PAD4-mediated histone citrullination is uncoupled from NETosis.

NETosis is induced by diverse stimuli. In this study, we focused on the fungal pathogen *C. albicans*, a prominent inducer of NETosis (13, 15), and we also included the non-microbial stimuli PMA and ionomycin. Because of the Ca^2+^-dependency of PAD4 activity (31), Ca^2+^ ionophores were postulated to act as bona fide stimuli for PAD4-mediated histone citrullination linked to subsequent chromatin release (53). In our hands, however, NET induction by ionomycin also remained unaffected by PAD4-inhibition. We, therefore, believe that the independence of NETosis from histone citrullination is broadly applicable, although we cannot exclude stimulus-dependent involvement of PAD4 in NETosis.

Diverse processes have been attributed to PAD4 activity beyond NETosis. Although histones were among the first substrates of PAD4 to be identified (55, 56), over 70 putative PAD4 substrates have been described to date (57, 58). PAD4 activity is a general hallmark of neutrophil activation. In addition to modifying histones, PAD4 citrullinates transcription factors including NFκB and E2F-1 for enhanced nuclear translocation or promoter binding, respectively, and increased cytokine production (59, 60). PAD4 can also modulate T cell-mediated immune responses (61, 62) and the activity of inflammatory mediators including cytokines, chemokines, and host defense proteins (63–66), among other reported functions. Any of these NET-independent activities of PAD4 may underlie the slight defect in fungal control that we observed in PAD4-deficient mice during systemic candidiasis.

The implication of citrullinated autoantigens including citrullinated histones in rheumatoid arthritis led to the idea that NETosis is a source of these autoantigens and contributes to disease pathogenesis (67–69). Based on the above considerations, however, the role of PAD4 in rheumatoid arthritis is more likely independent of NETosis. Likewise, the idea that deletion or inhibition of PAD4 improves disease symptoms in preclinical models of lupus, vasculitis, and glomerulonephritis due to inhibition of NETosis (70–72) calls for careful re-analysis. The pharmacological targeting of PAD4 in these diseases in human patients should be approached with care before fully understanding the pathomechanism in each condition. Moreover, another member of the peptidylarginine deiminase family, PAD2, can also catalyze protein citrullination in response to certain stimuli including TNF (73).

Citrullination of histones can easily be detected by immunohistochemistry or immunofluorescence on tissue sections. The postulated link between PAD4-mediated histone citrullination and NETosis introduced a welcome approach for easy identification of NETs and, consequently, this posttranslational modification is now commonly used as a proxy for NETs, especially in tissues *in vivo* (15, 30, 35, 73, 74). Based on our results, however, histone citrullination can no longer be accepted as a reliable marker for NETs.

Direct visualization of NETs remains difficult, especially in tissues where a high cell density and autofluorescence hinders the unambiguous extracellular localization of DNA and proteins. Accordingly, we were unable to detect extracellular chromosomal DNA in the *C. albicans*-infected kidney or tongue, both organs displaying a particularly high cell density and high autofluorescence. To overcome these tissue-specific limitations, most *in vivo* studies on NETs focused on the lung or the vasculature (13, 15, 75). However, these are not target organs of *C. albicans*. A specific marker for the visualization of NETs is still awaiting its discovery.

The lack of a specific marker for NETs has also hampered the assessment of the requirement of NETs in various conditions. PAD4-deficient mice were employed as a model of NET-deficiency to test the antimicrobial role of NETs in immune defense against various pathogenic microbes, including *Shigella flexneri*, group A *Streptococcus*, and influenza virus (30, 34, 35). Only in some cases an impairment in the protective response was observed, which was attributed to defects in NETosis (30), while susceptibility to infection was not generally affected by PAD4-deficiency (34, 35). Our findings put in question the interpretation of the results from studies with PAD4-deficient mice, as we show that NETosis occurs independently of PAD4.

Lytic and non-lytic forms of NETosis have been described. The latter is independent of NADPH oxidase and MPO and does not involve citrullination of histone H3 (76). Although we cannot formally exclude a non-lytic mechanism of NETosis as an alternative explanation for PAD4-independent NETosis in response to *C. albicans*, our data with neutrophils from acquired MPO-deficiency and the visualization of chromatin decondensation support a lytic process.

The distinction of NETosis from other forms of lytic cell death that are accompanied with the release of DNA into the extracellular space such as necroptosis, which can occur as a consequence of cellular damage, remains difficult (77). Bacteria such as *Staphylococcus aureus* can induce NET-like structures *via* pore-forming toxins that induce Ca^2+^ influx, citrullination, and chromatin extrusion (78, 79). Moreover, particles of different sizes and shapes have been shown to induce NET-like DNA release in association with neutrophil necroptosis, both in human and murine neutrophils (80). Whether *C. albicans*-induced NET-like structures result from cellular damage caused by hyphal virulence factors has not been fully clarified. *C. albicans*-secreted aspartyl proteinases seem to play an important role in triggering NETosis (81). Whether the recently identified pore-forming toxin Candidalysin, which can induce cellular damage in host cells (82), also contributes to NETosis has not been reported. While research on Candidalysin focused mainly on epithelial cells, Candidalysin may also cause damage in immune cells (83) including neutrophils. We found NETs to be induced by different isolates of *C. albicans* including strain 101, which expresses only very low levels of the Candidalysin-encoding gene *ECE1* and is unable to cause cellular damage (38). This result contradicts the idea that *C. albicans*-induced NETosis may be the result of toxin-induced cellular damage. More work is needed to identify the fungal determinants and the neutrophil molecular pathways sensing *C. albicans* for NET induction.

Together, our study confirms the efficient induction of NETosis by *C. albicans* hyphae, but it challenges the current view on the molecular mechanism of NET induction as well as the reliability of frequently used approaches in the field to document the generation and function of NETs *in vivo*.

## Ethics Statement

All mouse experiments described in this study were conducted in strict accordance with the guidelines of the Swiss Animal Protection Law and were performed under protocols approved by the Veterinary office of the Canton Zurich, Switzerland (license number 201/2012 and 183/2015). All efforts were made to minimize suffering and ensure the highest ethical and humane standards. Experiments with human blood samples were approved by the local ethics committee (KEK-ZH-NR: 2009-0062, BASEC-Nr. 2016-01908) and were performed and analyzed anonymously. Blood samples were obtained after informed consent and in accordance with the declaration of Helsinki principles.

## Author Contributions

EG and SL-L designed the experiments and wrote the manuscript. EG, CL, and NK performed experiments. EG, CL, NK, and SL-L analyzed data. ES performed transmission electron microscopy. AT provided peripheral blood samples from patients with acquired MPO-deficiency. SL-L supervised the project.

## Conflict of Interest Statement

The authors declare that the research was conducted in the absence of any commercial or financial relationships that could be construed as a potential conflict of interest.
