# Supplementary material for: Candida albicans-Induced NETosis Is Independent of Peptidylarginine Deiminase 4
Source: Front Immunol. 2018 Jul 9;9:1573. doi: 10.3389/fimmu.2018.01573 (PMC6046457; doi:10.3389/fimmu.2018.01573)
Supplement: Supplementary file 1 [file Data_Sheet_1.PDF]

Figure S1│ CitH3 occurs during systemic and oropharyngeal *C. albicans* infection

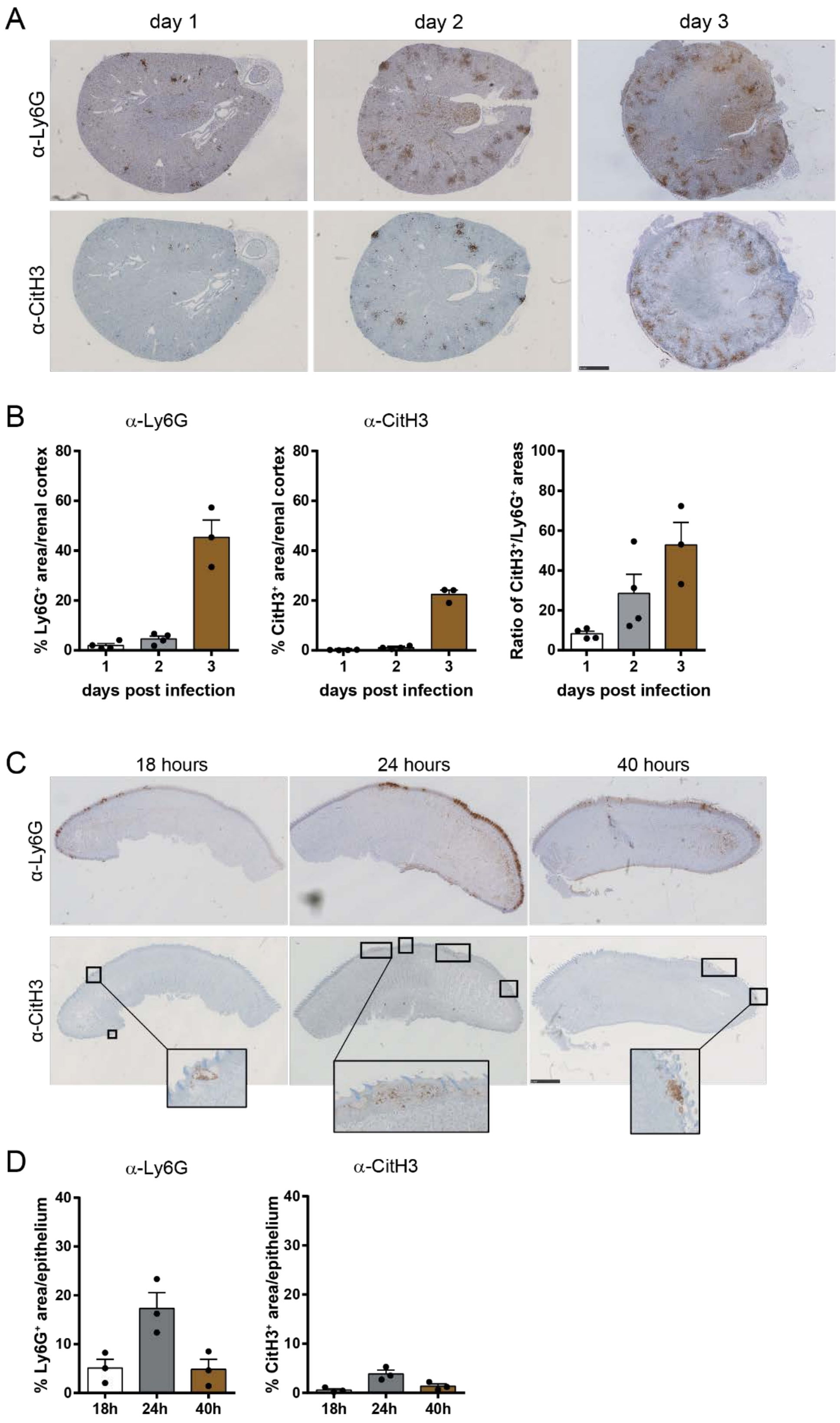

Figure S1│Citrullination of histone H3 occurs during systemic and oropharyngeal *C. albicans* infection. (A-B) Mice were infected with *C. albicans* intravenously and analyzed on day 1, day 2, or

day 3 post-infection. Consecutive kidney sections were stained with α-Ly6G for detection of neutrophils (A, top) or with α-CitH3 for histone citrullination (A, bottom). The percentage of Ly6G$^+$ or CitH3$^+$ areas in the renal cortex and the ratio of CitH3$^+$ to Ly6G$^+$ areas were determined in B. **(C-D)** Mice were infected with *C. albicans* sublingually and analyzed at 18 hours, 24 hours or 40 hours post-infection. Consecutive tongue sections were stained with α-Ly6G for detection of neutrophils (C, top) or with α-CitH3 for histone citrullination (C, bottom). The percentage of Ly6G$^+$ and CitH3$^+$ areas within the epithelium were determined in D. In B and D, each symbol represents an individual mouse and the mean with SEM of each group is indicated. Scale bar = 1 mm.

Figure S2│Gating strategies for kidney and tongue neutrophils

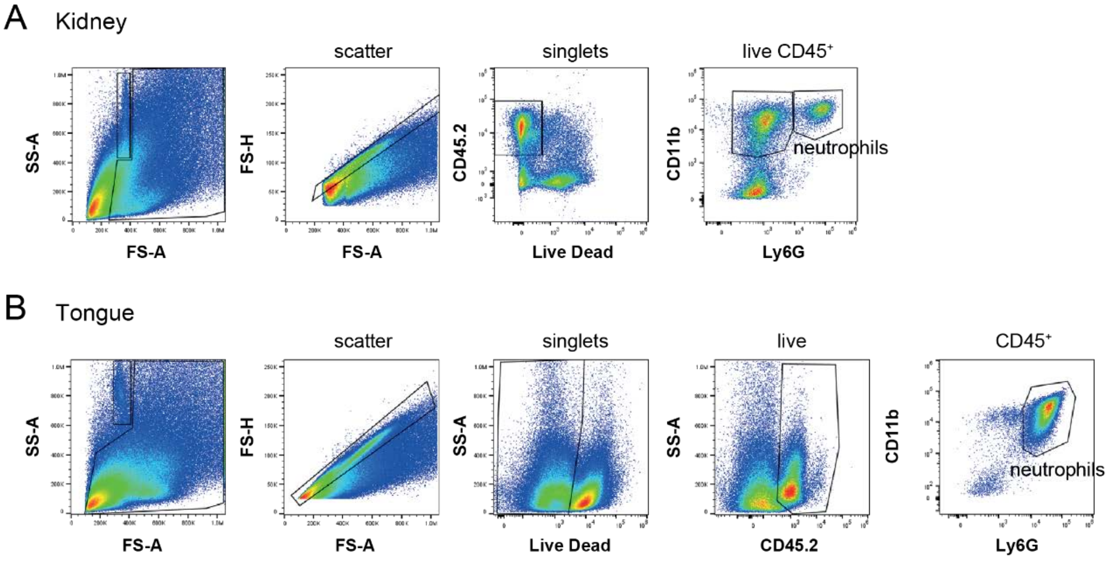

Figure S2│Gating strategies for kidney and tongue neutrophils. Representative FACS plots showing the analysis of Ly6G^{hi} CD11b^{+} neutrophils in the kidney (A) or tongue (B) of infected WT mice. Neutrophils were identified by pre-gating on singlets and CD45^{+} live cells.

Figure S3│Fungal control in PAD4-deficient mice is not dose dependent

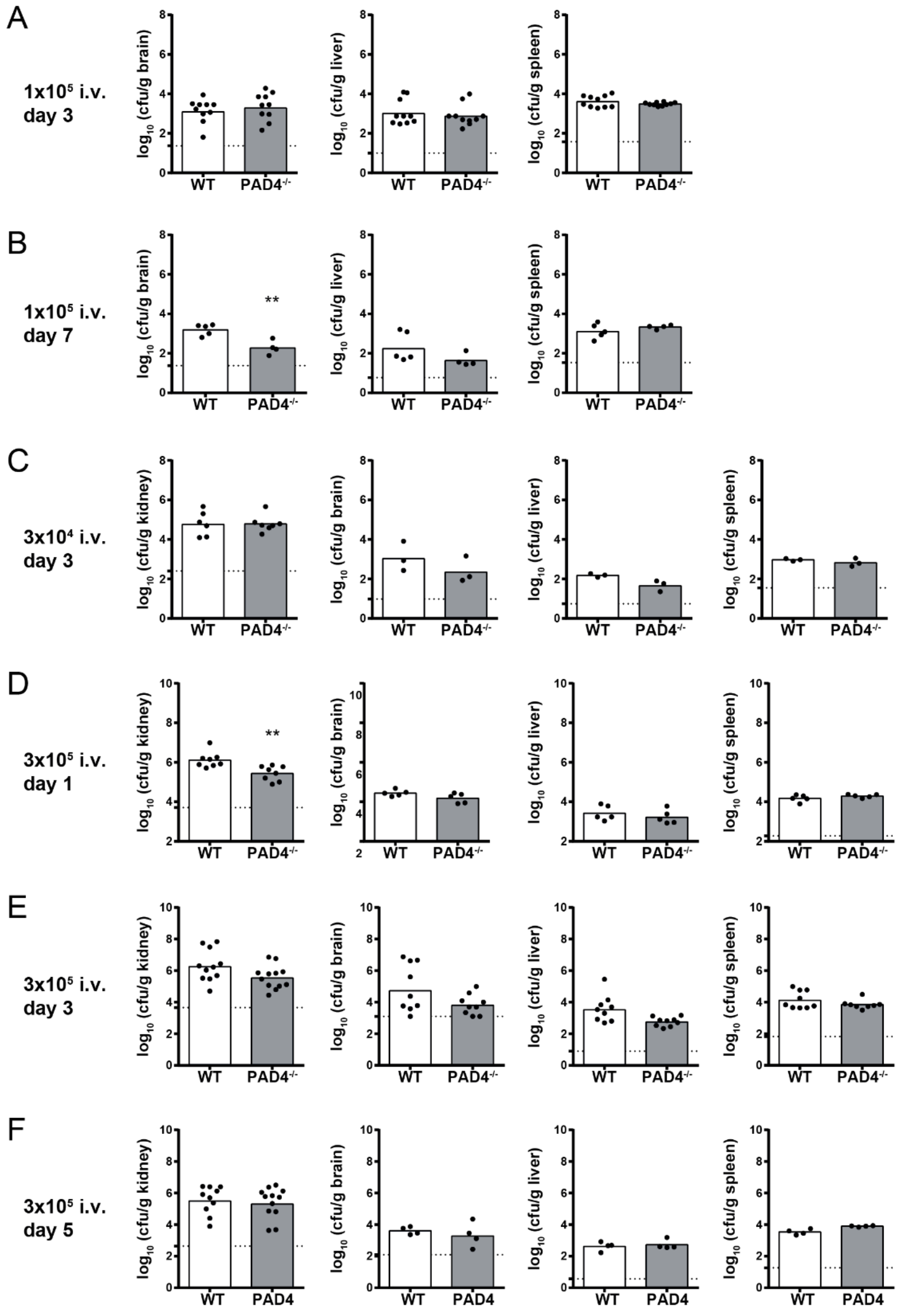

Figure S3│Fungal control in PAD4-deficient mice is not dose dependent. (A-B) WT and $PAD4^{-/-}$ mice were infected intravenously with 10^5 *C. albicans* yeast cells and the fungal burden was determined in the brain, liver, and spleen on day 3 (A) and day 7 post-infection (B). **(C)** WT and $PAD4^{-/-}$ mice were infected intravenously with 3x 10^4 *C. albicans* yeast cells and the fungal burden in the

kidney, brain, liver, and spleen was determined on day 3 post-infection. **(D-F)** WT and PAD4$^{-/-}$ mice were infected intravenously with 3x 10^5 *C. albicans* yeast cells and the fungal burden was determined in the kidney, brain, liver, and spleen on day 1 (D), day 3 (E), and day 5 post-infection (F). Each symbol represents an individual mouse, the lines represent the geometric mean of each group. The dotted lines represent the detection limit.
